# Deep Learning-Based Analytic Models Based on Flow-Volume Curves for Identifying Ventilatory Patterns

**DOI:** 10.3389/fphys.2022.824000

**Published:** 2022-01-28

**Authors:** Yimin Wang, Qiasheng Li, Wenya Chen, Wenhua Jian, Jianling Liang, Yi Gao, Nanshan Zhong, Jinping Zheng

**Affiliations:** State Key Laboratory of Respiratory Disease, National Center for Respiratory Medicine, National Clinical Research Center for Respiratory Disease, Guangzhou Institute of Respiratory Health, First Affiliated Hospital of Guangzhou Medical University, Guangzhou, China

**Keywords:** artificial intelligence, flow-volume curve, ventilatory pattern, pulmonary function testing, deep learning

## Abstract

**Introduction:**

Spirometry, a pulmonary function test, is being increasingly applied across healthcare tiers, particularly in primary care settings. According to the guidelines set by the American Thoracic Society (ATS) and the European Respiratory Society (ERS), identifying normal, obstructive, restrictive, and mixed ventilatory patterns requires spirometry and lung volume assessments. The aim of the present study was to explore the accuracy of deep learning-based analytic models based on flow–volume curves in identifying the ventilatory patterns. Further, the performance of the best model was compared with that of physicians working in lung function laboratories.

**Methods:**

The gold standard for identifying ventilatory patterns was the rules of ATS/ERS guidelines. One physician chosen from each hospital evaluated the ventilatory patterns according to the international guidelines. Ten deep learning models (ResNet18, ResNet34, ResNet18_vd, ResNet34_vd, ResNet50_vd, ResNet50_vc, SE_ResNet18_vd, VGG11, VGG13, and VGG16) were developed to identify patterns from the flow–volume curves. The patterns obtained by the best-performing model were cross-checked with those obtained by the physicians.

**Results:**

A total of 18,909 subjects were used to develop the models. The ratio of the training, validation, and test sets of the models was 7:2:1. On the test set, the best-performing model VGG13 exhibited an accuracy of 95.6%. Ninety physicians independently interpreted 100 other cases. The average accuracy achieved by the physicians was 76.9 ± 18.4% (interquartile range: 70.5–88.5%) with a moderate agreement (κ = 0.46), physicians from primary care settings achieved a lower accuracy (56.2%), while the VGG13 model accurately identified the ventilatory pattern in 92.0% of the 100 cases (*P* < 0.0001).

**Conclusions:**

The VGG13 model identified ventilatory patterns with a high accuracy using the flow–volume curves without requiring any other parameter. The model can assist physicians, particularly those in primary care settings, in minimizing errors and variations in ventilatory patterns.

## Introduction

Pulmonary function tests (PFTs) are integral to the diagnosis and monitoring of patients with respiratory abnormalities for pulmonologists, nurses, technicians, physiologists, and researchers (Liou and Kanner, 2009; Halpin et al., 2021). According to the guidelines set by the American Thoracic Society (ATS)/European Respiratory Society (ERS), a trained technician performs spirometry and a lung volume test to identify the ventilatory patterns as normal, obstructive, restrictive, or mixed patterns in consultation with a pulmonologist (Pellegrino et al., 2005).

Chronic respiratory diseases pose a threat to the Chinese population. Despite this knowledge, the use of PFTs is limited (Zhong et al., 2007; Wang et al., 2018; Huang et al., 2019). For an early and accurate detection of chronic respiratory disorders, PFTs, particularly spirometry, should be urgently employed across all levels of healthcare (CPC Central Committee State Council, 2016). A Belgian multicenter study demonstrated that pulmonologists could only reach an accuracy of 74.4% in identifying ventilatory patterns using PFTs according to the ATS/ERS guidelines (Topalovic et al., 2019). Therefore, fast and accurate interpretation of spirometry results is crucial in primary care settings, and novel interpretation approaches for ventilatory patterns are warranted.

Several software applications and algorithms established for interpreting PFTs have been investigated in healthcare research (Giri et al., 2021). A stacked autoencoder-based neural network has been used to detect abnormalities using spirometric parameters such as the forced expiratory volume in the first second (FEV_1_), forced vital capacity (FVC), FEV_1_/FVC, and flow–volume curves (Trivedy et al., 2019). Ventilatory patterns have a characteristic configuration in the flow–volume curves (Pellegrino et al., 2005). A study showed an accuracy of 97.6% when using flow–volume curves and artificial intelligence algorithms to identify normal and abnormal ventilatory patterns (Jafari et al., 2010). Moreover, some studies involving small sample size explored algorithms for PFT signal processing and classification (Veezhinathan and Ramakrishnan, 2007; Sahin et al., 2010; Nandakumar and Nandakumar, 2013). Topalovic et al. (2019) developed a model to recognize normal, obstructive, restrictive, and mixed ventilatory patterns based on spirometry and lung volume test results according to the ATS/ERS guideline. However, some algorithms failed to capture all the patterns and, therefore, could not be applied in clinical practice. Some modalities for ventilatory pattern identification required both spirometry and lung volume data; thus, they are limited by the fact that most primary care settings can only carry out spirometry.

The aim of the present study was to determine whether or not the deep learning-based analytic models could facilitate ventilatory pattern identification using flow–volume curves and outperform physicians. Another aim was to assess the accuracy and interrater variability of physicians in interpreting ventilatory patterns and to compare the accuracy of test reading by physicians at different levels of healthcare settings as well as with different work experiences and training.

## Materials and Methods

### Pulmonary Function Tests

**FIGURE 1:**
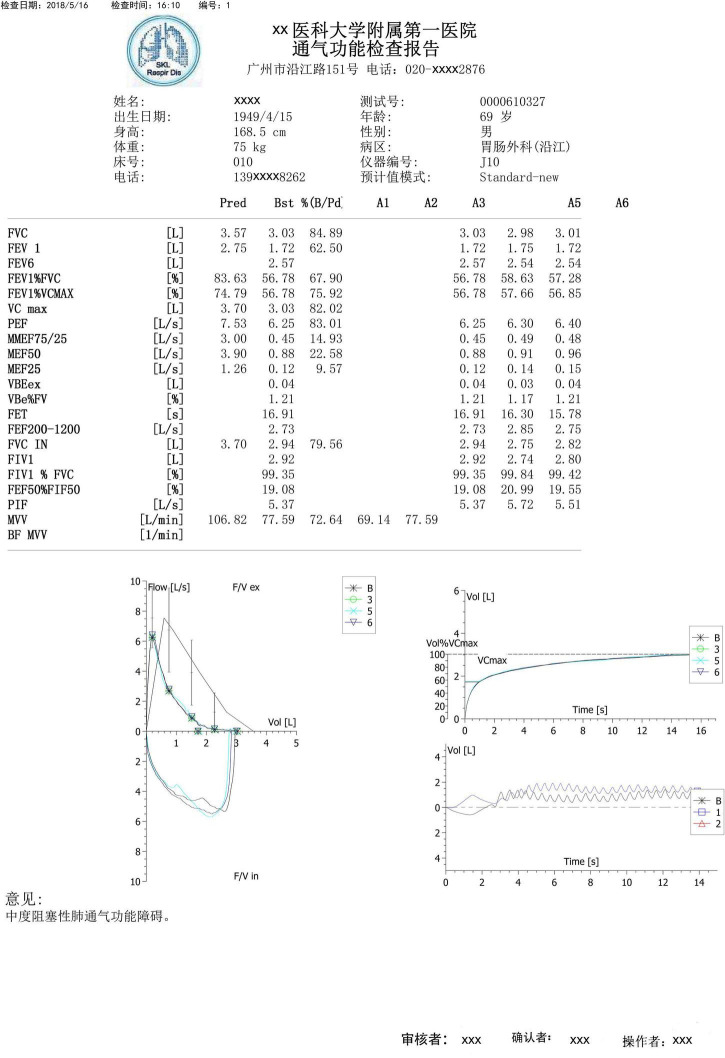
A typical example of a spirometry record. A typical spirometry record in a pdf includes parameters, flow–volume curves, and volume-time curves, which were obtained from devices. Example in the Chinese language.

Spirometry and lung volume tests were performed using the MasterScreen-Pneumo PC spirometer (Jaeger, Hochberg, Germany) and whole-body plethysmography (Jaeger, Hochberg, Germany), respectively. Trained technicians performed all the procedures, interpreted the results based on the ATS/ERS guidelines, and validated the results through expert opinion in daily work (Pellegrino et al., 2005; Graham et al., 2019). At least three acceptable maneuvers were needed. Spirometry parameters, flow–volume curves, and volume–time curves were obtained from the devices and converted to a fixed PDF format. Figure 1 illustrates a representative spirometry record. Flow–volume curves were displayed with 5 mm/L/s of flow and 2 L/s-to-1 L of the flow-to-volume ratio according to the ATS guidelines (Culver et al., 2017).

All the flow–volume curves without lung function parameters extracted from baseline spirometry records used for training, validating, and testing the deep learning-based models were acquired from the lung function laboratory of the First Affiliated Hospital of Guangzhou Medical University from October 2017 to October 2020. Further, 100 cases were achieved from the same laboratory in September 2017 to assess and compare the performance of the best-performing model with that of physicians. The inclusion criterion for spirometry records was the presence of at least one acceptable flow–volume curve, regardless of the patient’s age, sex, or ventilatory pattern.

### Physicians’ Selection

The physicians who participate in this study were from healthcare settings equipped with lung function laboratories and had routinely performed PFTs. The inclusion criterion was daily involvement in the operation and interpretation of PFTs. One physician, willing to participate in the current study, was randomly selected from each hospital regardless of the work experience, presence/absence of training, or hospital level.

### Study Design

**FIGURE 2:**
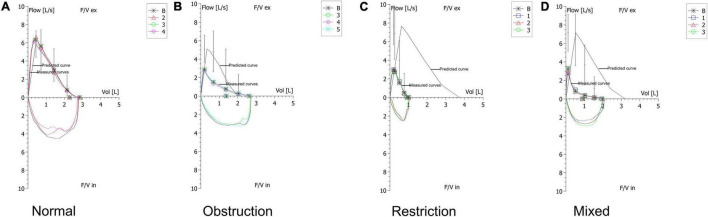
Typical examples of ventilatory patterns of spirometry. **(A)** Example of a normal pattern. **(B)** Example of an obstructive pattern that shows a concave shape on the expiratory flow. **(C)** Example of a restrictive pattern that shows a convex shape on the expiratory flow. **(D)** Example of a mixed pattern that shows characteristics of coexistence of obstruction and restriction.

Ten deep learning-based models were developed using only spirometric flow–volume curves. Figure 2 illustrates representative examples of ventilatory patterns identified using spirometry. The performance of the best-performing model was compared with that of physicians, who independently interpreted 100 PFT records, including lung function parameters, flow–volume curves, and volume-time curves, and answered a questionnaire at the online *WenJuanXing* platform (China)^1^ within 3 weeks. The flow–volume curves of the same cases were evaluated by the best-performing model.

### Model Development

The deep learning-based models for automated interpretations were developed using Python version 3.7.6, combined with deep learning framework PaddlePaddle version 1.8^2^ and its image recognition toolset PaddleClas.^3^ PaddleClas is used in industries and academia and contains various mature deep learning algorithm models. Ten classic image recognition models, including ResNet18 (He et al., 2016), ResNet34 (He et al., 2016), ResNet18_vd (He et al., 2019), ResNet34_vd (He et al., 2019), ResNet50_vd (He et al., 2019), ResNet50_vc (He et al., 2019), SE_ResNet18_vd (Jie et al., 2018), VGG11 (Simonyan and Zisserman, 2014), VGG13 (Simonyan and Zisserman, 2014), and VGG16 (Simonyan and Zisserman, 2014) were developed to complete the classification tasks from the model library in PaddleClas.

A total of 18,909 baseline spirometry records, including 9,598 normal, 4,420 obstructive, 2,704 restrictive, and 2,187 mixed patterns, were used to develop the models. A stratified random sampling method was used, and each pattern was distributed among the training, validation, and test sets at the ratio of 7:2:1. Table 1 shows the details of the datasets.

**TABLE 1:** The distribution of datasets to develop deep learning models.

Patterns	Training set	Validation set	Test set	Total
Normal	6,720	1,919	959	9,598
Obstruction	3,094	884	442	4,420
Restriction	1,894	540	270	2,704
Mixed	1,532	437	218	2,187
Total	13,240	3,780	1,889	18,909

The original spirometry records were stored in the PDF format in color. For subsequent data processing, the original spirometry records were converted to the PNG format in color. Subsequently, the flow–volume curve images including the predicted and measured curves were extracted from the spirometry records with a pixel size of 328 × 244 using PyMuPDF version 1.18.15. Figure 2 shows the extracted flow-volume curves with the red, green, and blue channels.

The order of the training, validation, and test sets with the labels was randomized and then arranged in separate lists. The parameters in each selected PaddleClas model configuration file were customized. The shape of image was set to (3, 224, 224). The number of classes was set to four. The appropriate training batch size was selected according to the size of the graphics processing unit (GPU) memory. The number of training epochs was set to 90. Finally, the other settings were set to default. The lists of the training and validation sets were used for model training using the Nvidia RTX 2060 super GPU workstation. After the training process, the optimal model was selected according to the best average accuracy on the test set.

### Statistical Analysis

The gold standard for pattern classifications followed the ATS/ERS guidelines (Pellegrino et al., 2005). The Kruskal-Wallis test was performed for inter-group comparisons. The one-sample *t*-test was performed to identify the difference between the selected model and physicians’ performances. Fleiss’ Kappa was used to measure inter-observer agreements in pattern identification. The performance of models was tested using the confusion matrixes in Scikit-learn version 0.22.1^4^ of Python version 3.7.4. The receiver operating characteristic curve was analyzed using Scikit-learn and Matplotlib version 3.1.3,^5^ with the “micro” and “macro” parameters (Fawcett, 2006) were set by One-vs-one algorithm (Hand and Till, 2001) and One-vs-rest algorithm (Provost and Domingos, 2001), respectively. Other statistical analyses were performed with SPSS version 26.0.

## Results

### Study Population

Ninety physicians interpreted the 100 PFT records and produced 9,000 evaluations for ventilatory pattern identification. They came from tertiary hospitals (*n* = 43), secondary hospitals (*n* = 25), and primary care settings (*n* = 22) of 18 Chinese provinces (or equivalent) around mainland China. Among them, 30.0% (*n* = 27), 24.4% (*n* = 22), and 45.6% (*n* = 41) had <1, 1–3, and >3 years of work experience, respectively. In addition, previously trained physicians (*n* = 63) who had attended standardized PFT training sponsored by the Chinese Thoracic Society were significantly more in number than those who had not been trained (*n* = 27). Regarding the characteristics of the 100 PFT records, there were 44 normal, 18 obstructive, 23 restrictive, and 15 mixed patterns (Table 2).

**TABLE 2:** Characteristics of the 100 cases at study baseline.

Parameters	Normal	Obstruction	Restriction	Mixed
Reports, N	44	18	23	15
Sex, M/F	21/23	13/5	14/9	13/2
Age, years	59.5 (51.3–63.0)	55.0 (37.0–62.3)	49.0 (31.0–65.0)	67.0 (63.0–70.0)
FEV_1_% pred, %	98.9 (89.6–109.1)	75.9 (63.6–88.1)	74.0 (56.4–77.5)	41.3 (29.8–49.3)
FVC% pred, %	102.1 (94.2–111.3)	92.6 (87.2–113.4)	73.7 (53.9–77.8)	68.2 (44.8–72.8)
FEV_1_/FVC ratio	0.79 (0.75–0.82)	0.67 (0.57–0.71)	0.86 (0.79–0.89)	0.54 (0.46–0.60)
FEF_50%_ % pred, %	77.6 (59.9–94.3)	34.9 (23.1–56.5)	60.0 (49.5–80.9)	12.9 (9.7–16.6)
FEF_75%_ % pred, %	57.2 (40.1–72.5)	26.6 (17.1–39.9)	48.1 (36.8–71.4)	13.9 (11.2–19.2)
MMEF_%_ pred, %	68.0 (54.0–82.0)	31.3 (20.0–50.0)	57.4 (40.7–72.2)	12.3 (9.1–15.6)

*Data were shown in Median (interquartile range). FEV_1_, forced expiratory volume in one second; FVC, forced vital capacity; FEF_x%_, flow at x% FVC; MMEF, maximal mid expiratory flow; % pred, % predicted.*

### Model Performances

**FIGURE 3:**
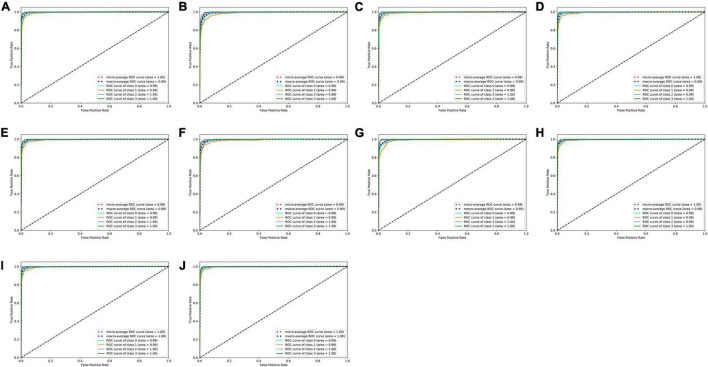
ROC curves of ten deep learning-based models. **(A–J)** ROC curves of ResNet18, ResNet34, ResNet18_vd, ResNet34_vd, ResNet50_vd, ResNet50_vc, SE_ResNet18_vd, VGG11, VGG13, and VGG16 models to classify types of ventilatory patterns, respectively. Class 0 = normal; Class 1 = obstruction; Class 2 = restriction; Class 3 = mixed; ROC = receiver operating characteristic.

On the test set, the 10 deep learning-based analytic models based on the flow–volume curves identified ventilatory patterns with an average accuracy ranging from 92.7 to 95.6%. The models identified the obstructive ventilatory pattern with a lower accuracy between 86.2 and 92.3%. Further analysis of the degree of severity of these incorrectly identified obstructive cases, the mild cases were the most difficult to identify, which were incorrectly identified as normal cases (80.3–91.8%). The best-performing model was VGG13 with the highest average accuracy. Table 3 and Figure 3 show the details of the model performance. The model required <1 s to assess the ventilatory pattern from each spirometry record.

**TABLE 3:** The performance of 10 deep learning-based models in the test set.

	Accuracy of pattern classifications				
Models	Normal (%)	Obstruction (%)	Restriction (%)	Mixed (%)	Average accuracy (%)
ResNet18	98.3	88.2	94.8	97.3	94.7
ResNet34	97.0	87.1	94.8	91.7	92.7
ResNet18_vd	98.03	88.9	94.1	95.4	94.2
ResNet34_vd	98.5	90.3	95.2	95.9	95.0
ResNet50_vd	98.8	87.3	94.8	95.4	94.1
ResNet50_vc	98.1	86.2	93.0	96.3	93.4
SE_ResNet18_vd	97.7	86.9	96.3	93.6	93.6
VGG11	98.2	91.0	96.7	94.5	95.1
VGG13	97.7	91.0	96.7	97.3	95.6
VGG16	98.4	92.3	95.9	95.0	95.4

When evaluating the 100 cases, the VGG13 model classified ventilatory patterns with an average accuracy of 92.0%. The restrictive pattern was more difficult (sensitivity: 87%) to identify compared to other patterns but was identified with a perfect specificity of 100%. Moreover, the model incorrectly classified three normal patterns as obstructive patterns and one obstructive pattern as a normal pattern. Table 4 shows performance of VGG13 in identifying the ventilatory pattern of 100 cases according to the confusion matrix.

**TABLE 4:** Confusion matrix shows the performance of the VGG13 model at interpreting ventilatory patterns in 100 cases.

		VGG13 model’ evaluation			
	Normal	Obstruction	Restriction	Mixed	Total
Normal	**41**	3	0	0	44
Obstruction	1	**17**	0	0	18
Restriction	2	0	**20**	1	23
Mixed	0	1	0	**14**	15
	
Total	44	21	20	15	100
Sensitivity,%	93.2	94.4	87.0	93.3	
Specificity,%	94.6	95.1	100.0	98.8	
PPV,%	93.2	81.0	100.0	93.3	
NPV,%	94.6	98.7	96.3	98.8	

*Data are presented as n, unless otherwise stated. The bold values mean the true positive for each pattern.*

*PPV, positive predictive value; NPV, negative predictive value.*

### Physicians’ Performances

The ventilatory pattern evaluated by physicians accurately followed the guidelines in 76.9 ± 18.4% cases (interquartile range: 70.5–88.5%). The physicians from primary care settings achieved an accuracy of 56.2 ± 21.6% (interquartile range: 34.0–76.3%). The most difficult pattern to identify was the restrictive pattern (sensitivity: 70.0%), which was mostly incorrectly classified as the mixed pattern (*n* = 329). In addition, 724 normal patterns were incorrectly classified as obstructive pattern, and 304 obstructive patterns were incorrectly classified as normal patterns. Table 5 demonstrates the performance of physicians according to the confusion matrix. The interrater disagreement among physicians identifying the ventilatory patterns was a κ of 0.46.

**TABLE 5:** Confusion matrix shows the performance of 90 physicians at interpreting ventilatory patterns in 100 cases.

		90 physicians’ evaluation				
	Normal	Obstruction	Restriction	Mixed	Total	N subjects
Normal	**3,115**	724	73	48	3,960	44
Obstruction	304	**1,203**	42	71	1,620	18
Restriction	202	90	**1,449**	329	2,070	23
Mixed	68	166	41	**1,075**	1,350	15
		
Total	3,689	2,183	1,605	1,523	9,000	
Sensitivity%	78.7	74.3	70.0	79.6		
Specificity%	88.6	86.7	97.7	94.1		
PPV%	84.4	55.1	90.3	70.6		
NPV%	84.1	93.9	91.6	96.3		

*Data are presented as n, unless otherwise stated. The bold values mean the true positive for each pattern. Abbreviations see in Table 4.*

**FIGURE 4:**
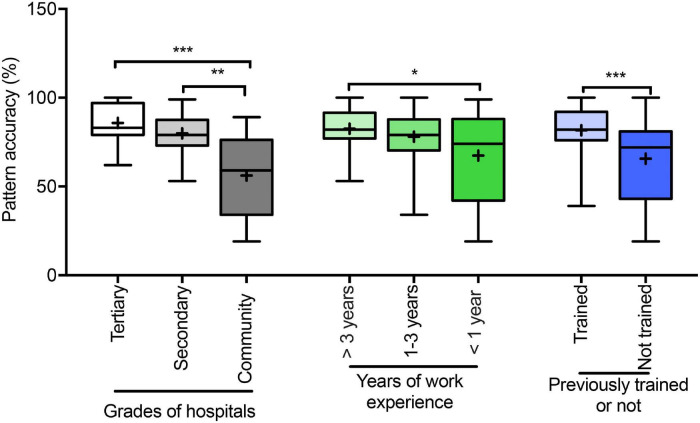
Accuracy (%) of ventilatory pattern evaluations of physicians. Accuracy (%) of pattern evaluations of physicians belong to different grades of hospitals; different years of work experience, and presence/absence training. Box-and-whisker plots show median with interquartile range (box) and range (whiskers); the mean is indicated by “+”; *P < 0.05, **P < 0.001, ***P < 0.0001.

Regarding the performance of pulmonologists compared across hospital levels, years of work experience, and presence/absence of training, significant differences were found between tertiary hospitals and community settings (*P* < 0.0001), work experience of >3 years and <1 year (*P* < 0.05), and presence and absence of training (*P* < 0.0001; Figure 4).

### Comparison of VGG13 With Physicians

**FIGURE 5:**
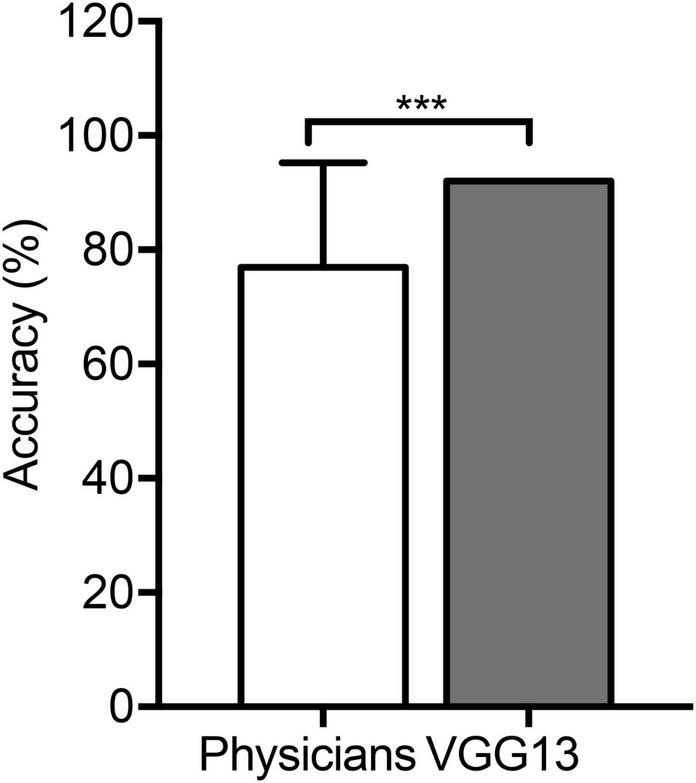
Comparison in the VGG13 model and physicians. An average accuracy (%) of pattern identification between the VGG13 model and 90 physicians. ***P < 0.0001.

The VGG13 model correctly identified the ventilatory pattern using flow–volume curves at a significantly higher accuracy compared to the physicians (92.0 vs. 76.9%) who had identified patterns according to the ATS/ERS guidelines (*P* < 0.0001, Figure 5), although the sensitivity and the positive predictive value showed the same trends (Tables 4, 5).

## Discussion

In the current study, the 10 deep learning-based analytic models based on flow-volume curves were developed to identify ventilatory patterns. The best-performing model, VGG13, showed an average accuracy of 95.6% on the test set. The accuracy and consistency in performance of the VGG13 model and physicians were compared for the ventilatory pattern identification of 100 other cases. The VGG13 model identified ventilatory patterns with high accuracy (92.0%) and efficiency (<1 s/record), while physicians accurately identified ventilatory patterns according to the guidelines with a relatively low accuracy (76.0%) and a κ of 0.46. Further, primary care physicians achieved an even lower accuracy (56.2%).

Automated algorithms to detect spirometric abnormalities have been studied previously. These algorithms exploited features extracted from spirometric parameters and spirogram (Asaithambi et al., 2012; Ioachimescu and Stoller, 2020). Ioachimescu and Stoller (2020) used an alternative parameter (area under the expiratory flow–volume curve) to differentiate normal, obstructive, restrictive, and mixed patterns. When a machine learning algorithm used this novel parameter in combination with FEV_1_, FVC, and FEV_1_/FVC *z*-scores, the patterns could be differentiated appropriately. Conversely, our proposed model used only flow–volume curves based on display characteristics of patterns instead of parameters to classify the pattern. Asaithambi et al. (2012) classified normal and abnormal respiratory functions using a neuro-fuzzy based on spirometry parameters, such as FEV_1_, FVC, and peak expiratory flow, obtained from 250 subjects at an accuracy of 97.5%. The models developed in the present study were based on a larger study population, identified all four patterns, and provided stable performances while processing large spirometry datasets. Therefore, these models could not only be used in routine clinical practice but also help deal with large spirometric data in research.

PFTs are routinely interpreted by physicians to diagnose respiratory abnormalities. Interpretive strategies require both spirometry and lung volume assessments. In our study, physicians from tertiary hospitals, who worked in the typical university centers responsible for teaching medical students, could not reach perfect accuracy in pattern identification. Primary care physicians performed with a lower accuracy probably because most primary care centers do not have lung volume measurement devices and are equipped only with spirometers. The lack of lung volume measurement devices may impede the use of PFTs in primary care settings. Furthermore, physicians with >3 years of work experience outperformed those with <1 year of work experience, thus suggesting that the performance of physicians was associated with their work experience. Our study further compared the correct identification of patterns between previously trained and untrained physicians. Those who had been trained performed significantly better than those who had not been trained. In summary, the performance of physicians interpreting spirometry depends on the working experience, prior training, and good platforms (Represas-Represas et al., 2013; Charron et al., 2018). In contrast, our model exhibited fast and stable performance that did not require much experience or training.

Compared to other patterns, the restrictive pattern was more difficult to identify for both the VGG13 model and physicians, which may be due to the fact that the flow–volume curves of this pattern are similar to those of the normal pattern. However, on the test set, the mild obstructive pattern was the most difficult to identify by any deep learning model and was incorrectly identified as a normal pattern. The obstructive pattern was also not easy for the physicians to identify. In contrast, the model obtained a much higher accuracy of 94.4% in identifying this pattern.

Despite the model showing good efficiency and accuracy, it had some limitations. It could handle large datasets but failed to identify the quality of spirometry. All test cases had acceptable curves, but in clinical settings, technicians perform quality control through visual inspection of curves and also in combined with measured values (Miller et al., 2005; Graham et al., 2019). Moreover, the spirometry records we used to develop the model were obtained exclusively from the Chinese population. Considering that normal spirometric values and curves differ among Asian, Caucasian, and African populations, our model may be not applicable to other ethnicities. However, we speculate that it could perform similarly if trained with datasets of other ethnicities, since the displays of flow–volume curves from ventilatory patterns are similar across ethnicities. Additionally, we only explored the conventional patterns. Specific patterns, such as upper airway obstruction (Fiorelli et al., 2019), “saw-tooth sign” (Bourne et al., 2017), and the “small-plateau sign” (Wang et al., 2021), require the recognition of flow–volume curves, including inspiratory and expiratory phases.

The best model VGG 13 completed the pattern identification task significantly better than the physicians from primary care settings. The model performed the task using only flow–volume curves obtained from the spirometry, whereas physicians needed to perform lung volume tests in addition. For clinical applications in the future, the model could be embedded into the software of different devices to help physicians in their routine work. Further, a cloud-based artificial intelligence system could be established to connect the devices from primary care settings to help general practitioners identify the ventilatory patterns from spirometry records in real time. However, the model was not trained to identify the quality of the spirometry. Therefore, a prerequisite for the correct functioning of the model is the need to ensure that spirometry respects internationally accepted quality criteria, which means that its use does not dispense that a trained technician performs spirometry with good quality.

## Conclusion

The proposed deep learning-based analytic model using flow–volume curves improved the detection accuracy of ventilatory patterns obtained from spirometry with high coherence and efficiency. In comparison, physicians, particularly those from primary care settings, were insufficiently trained in interpreting PFTs to identify ventilatory patterns. The deep learning model may serve as a supporting tool to assist physicians in identifying ventilatory patterns.

## Data Availability Statement

The original contributions presented in the study are included in the article/supplementary material, further inquiries can be directed to the corresponding authors.

## Ethics Statement

The studies involving human participants were reviewed and approved by the Ethics Committee of the First Affiliated Hospital of Guangzhou Medical University. Written informed consent for participation was not required for this study in accordance with the national legislation and the institutional requirements.

## Author Contributions

YW, WJ, JL, YG, and JZ: study design and hypothesis generation. YW, WC, WJ, and QL: data acquisition, analysis, or interpretation. YW, QL, NZ, and JZ: chart review and manuscript preparation. JZ and NZ: critical revision. JZ and YG: funding obtained. All authors listed approved this work for publication.

## Conflict of Interest

The authors declare that the research was conducted in the absence of any commercial or financial relationships that could be construed as a potential conflict of interest.

## Publisher’s Note

All claims expressed in this article are solely those of the authors and do not necessarily represent those of their affiliated organizations, or those of the publisher, the editors and the reviewers. Any product that may be evaluated in this article, or claim that may be made by its manufacturer, is not guaranteed or endorsed by the publisher.

## Footnotes

1
https://www.wjx.cn

2
https://gitee.com/paddlepaddle

3
https://gitee.com/paddlepaddle/PaddleClas

4
https://scikit-learn.org/

5
https://matplotlib.org/

## Abbreviations

FEV_1_: Forced expiratory volume in 1s
FVC: Forced vital capacity
PFT: pulmonary function testing.
